# Disruption of the N^α^-Acetyltransferase NatB Causes Sensitivity to Reductive Stress in *Arabidopsis thaliana*

**DOI:** 10.3389/fpls.2021.799954

**Published:** 2022-01-03

**Authors:** Monika Huber, Laura Armbruster, Ross D. Etherington, Carolina De La Torre, Malcolm J. Hawkesford, Carsten Sticht, Daniel J. Gibbs, Rüdiger Hell, Markus Wirtz

**Affiliations:** ^1^Centre for Organismal Studies, Molecular Biology of Plants Group, Heidelberg University, Heidelberg, Germany; ^2^School of Biosciences, University of Birmingham, Edgbaston, United Kingdom; ^3^Institute of Clinical Chemistry, NGS Core Facility, Medical Faculty Mannheim of Heidelberg University, Heidelberg, Germany; ^4^Rothamsted Research, Harpenden, United Kingdom

**Keywords:** N-terminal acetylation, co-translational modification, N-acetyltransferase NatB, ER-associated degradation, DOA10, proteostasis, reductive stress, N-degron

## Abstract

In *Arabidopsis thaliana,* the evolutionary conserved N-terminal acetyltransferase (Nat) complexes NatA and NatB co-translationally acetylate 60% of the proteome. Both have recently been implicated in the regulation of plant stress responses. While NatA mediates drought tolerance, NatB is required for pathogen resistance and the adaptation to high salinity and high osmolarity. Salt and osmotic stress impair protein folding and result in the accumulation of misfolded proteins in the endoplasmic reticulum (ER). The ER-membrane resident E3 ubiquitin ligase DOA10 targets misfolded proteins for degradation during ER stress and is conserved among eukaryotes. In yeast, DOA10 recognizes conditional degradation signals (Ac/N-degrons) created by NatA and NatB. Assuming that this mechanism is preserved in plants, the lack of Ac/N-degrons required for efficient removal of misfolded proteins might explain the sensitivity of NatB mutants to protein harming conditions. In this study, we investigate the response of NatB mutants to dithiothreitol (DTT) and tunicamycin (TM)-induced ER stress. We report that NatB mutants are hypersensitive to DTT but not TM, suggesting that the DTT hypersensitivity is caused by an over-reduction of the cytosol rather than an accumulation of unfolded proteins in the ER. In line with this hypothesis, the cytosol of NatB depleted plants is constitutively over-reduced and a global transcriptome analysis reveals that their reductive stress response is permanently activated. Moreover, we demonstrate that *doa10* mutants are susceptible to neither DTT nor TM, ruling out a substantial role of DOA10 in ER-associated protein degradation (ERAD) in plants. Contrary to previous findings in yeast, our data indicate that N-terminal acetylation (NTA) does not inhibit ER targeting of a substantial amount of proteins in plants. In summary, we provide further evidence that NatB-mediated imprinting of the proteome is vital for the response to protein harming stress and rule out DOA10 as the sole recognin for substrates in the plant ERAD pathway, leaving the role of DOA10 in plants ambiguous.

## Introduction

As sessile organisms, plants must continuously adapt to an ever-changing environment. One of the most rapid and efficient adaptive responses is the stress-induced activation of protein modification systems. These protein modifiers attach ubiquitin, phosphate, methyl groups, or many others and thereby adjust key protein characteristics, such as turnover, activity, or localization, within the cell (Kim et al., 2016). In the last decade, N^α^-terminal acetylation (NTA) has emerged as important mediator of protein fate. The transfer of an acetyl moiety from acetyl-CoA to the N-terminus of a protein is conserved throughout all domains of life and has the potential to alter protein-protein interactions, as well as the subcellular localization, folding, aggregation, and degradation of proteins (Aksnes et al., 2016). In eukaryotes, NTA ranges among the most abundant protein modifications, affecting 60% of the yeast proteome as well as 80–90% of soluble proteins in humans and plants (Arnesen et al., 2009; Bienvenut et al., 2012). NTA occurs co- and posttranslationally and is catalyzed by a set of specialized N-terminal acetyltransferases (Nats). Since the composition and substrate specificity of the five co-translationally acting Nats (NatA-E) in the cytosol is largely conserved among higher eukaryotes, it was long assumed that Nat diversification had not occurred in eukaryotic organisms (Rathore et al., 2016). The identification of a highly diversified posttranslational N^α^-acetyltransferase (Nat) machinery in plants has called this dogma into question (Giglione and Meinnel, 2021). Recent studies characterized a family of plant-specific posttranslationally acting Nats with dual-lysine acetyltransferase and N^α^-terminal acetyltransferase activity in the chloroplasts of the model plant *Arabidopsis thaliana* (Dinh et al., 2015; Koskela et al., 2018; Bienvenut et al., 2020). At least one of these plastidic Nats is required for the dynamic reorganization of thylakoid protein complexes during light stress-induced state transitions (Koskela et al., 2018). The diversification of the posttranslational plant Nat machinery during the evolution of eukaryotes is further evidenced by the diverging localization and concomitant function of the acetyltransferase NatF in humans and plants. While human NatF localizes to the Golgi membrane and is required to maintain Golgi integrity (Aksnes et al., 2015), plant NatF is plasma membrane resident and mediates the response to protein harming conditions like high salinity (Linster et al., 2020).

The majority (>70%) of eukaryotic proteins, however, is co-translationally acetylated by the evolutionary conserved ribosome-associated NatA-E complexes as soon as the nascent polypeptides emerge from the ribosome exit tunnel. In consequence, NTA has long been considered a static nonregulated protein modification. In Arabidopsis, the dominant Nat complexes NatA and NatB acetylate approximately 50 and 25% of soluble cytosolic proteins, respectively, (Linster and Wirtz, 2018). Whereas NatA targets N-termini which have undergone removal of the initiator methionine (iMet) by methionine aminopeptidases and hence start with A, S, T, C, V, and G (Linster et al., 2015), NatB acts on the iMet of N-termini starting with MD, ME, MN, and MQ (Huber et al., 2020). NatA and NatB have recently been implicated in the dynamic regulation of plant stress responses, challenging the view of NTA as static protein modification in plants (Linster et al., 2015; Xu et al., 2015; Huber et al., 2020). While a complete knockout of the NatA catalytic subunit NAA10 or the ribosome-anchoring subunit NAA15 is embryo lethal, a knockdown of any of the two subunits results in reduced plant growth as well as drought tolerance in *Arabidopsis thaliana*. This drought tolerance is only observed in NatA, but not NatB mutants, demonstrating a specific function of NatA-mediated proteome imprinting during drought stress. In line with these findings, the drought stress-induced phytohormone abscisic acid (ABA) regulates NatA abundance and in consequence the NTA status of plant proteins, providing the first evidence of hormonal control of NTA (Linster et al., 2015). This dynamic control of NTA might constitute an adaptation to the sessile lifestyle of plants that forces them to cope with highly variable and intense environmental stresses (Linster and Wirtz, 2018).

Like NatA, the NatB complex mediates plant development and a variety of stress responses. Depletion of the catalytic (NAA20) or the ribosome-anchoring subunit (NAA25) of the NatB complex leads to defects in embryo development and a substantial growth retardation (Ferrández-Ayela et al., 2013; Huber et al., 2020). NatB-dependent NTA was reported to stabilize individual stress-related proteins in plants. Those include the transcriptional regulator Sigma Factor Binding Protein 1 (SIB1) and various amincyclopropane-1-carboxylate oxidases (ACOs). While SIB1 is involved in the response to the defense hormone salicylic acid (Li et al., 2020), ACOs catalyze the biosynthesis of the phytohormone ethylene (Liu et al., 2021). Moreover, NatA and NatB antagonistically regulate the half-life of the Nod-like immune receptor Suppressor of NPR1 Constitutive 1 (SNC1). While NatA targets the dominant MMD-SNC1 variant and destabilizes the immune receptor upon acetylation, NatB-mediated acetylation has a stabilizing effect on the alternatively translated MD-SNC1 variant. By mediating the turnover of SNC1, NatA and NatB contribute to the control of the defense response against the parasitic oomycete *Hyaloperonospora arabidopsidis* Noco2 (Xu et al., 2015).

In addition to stabilizing specific stress-related proteins, NatB is involved in the adaptation to protein harming conditions like high salt and osmotic stress (Huber et al., 2020). High salinity and osmolarity impair the proper folding of proteins in the cytosol and the endoplasmic reticulum (ER). The accumulation of misfolded proteins in the ER is referred to as ER stress and triggers the unfolded protein response (UPR) which is highly conserved among eukaryotes (Liu et al., 2007b; Liu and Howell, 2010; Liu et al., 2011). Upon induction of the UPR, the expression of chaperones is stimulated and the translation of secretory and transmembrane proteins is attenuated to lighten the load of toxic misfolded proteins in the ER (Liu et al., 2007a; Che et al., 2010; Deng et al., 2011; Nagashima et al., 2011). Furthermore, misfolded proteins which cannot be rescued by chaperones are eliminated by the ER-associated protein degradation (ERAD) machinery (Strasser, 2018). The E3-ubiquitin ligase complexes HRD1 and DOA10 recognize ERAD substrates and ubiquitinate these misfolded proteins after their translocation into the cytosol to target them for degradation via the ubiquitin-proteasome system (UPS) in yeast and animals (Bays et al., 2001; Swanson et al., 2001).

So far, the mechanism by which NatB impacts the response to protein harming stress in plants is unknown. In yeast, two alternative hypotheses regarding the role of NatB in the degradation of unfolded proteins are controversially discussed. On the one hand, NatA and NatB create conditional degradation signals termed Ac/N-degrons. These acetylation marks are masked by correct folding of the Nat substrates. Only in unfolded proteins, these degrons become accessible and specifically recognized by the ER-associated E3-ubiquitin ligase DOA10, which targets proteins for degradation via the proteasome (Hwang et al., 2010; Shemorry et al., 2013). On the other hand, NatB-mediated NTA stabilizes various proteins of the ERAD machinery, including the HRD1 component DERLIN1 and the proteasome subunits RPT3, RPN11, and PRE1/β4. By stabilizing these ERAD effectors, NatB indirectly promotes the degradation of UPR targets (Zattas et al., 2013). Both hypotheses imply that a lack of NatB-mediated NTA leads to the accumulation of toxic, misfolded proteins in yeast, either because they are not recognized by the ERAD system (Hwang et al., 2010) or because the ERAD effectors themselves are unstable (Zattas et al., 2013). Since the ER-associated E3-ligases DOA10 and HRD1 are conserved in plants (Doblas et al., 2013; Chen et al., 2016), both findings suggest that plant NatB might be involved in stress-induced ERAD.

In this study, the effect of NatB-mediated NTA on ER stress induced by either dithiothreitol (DTT) or tunicamycin (TM) was investigated (Martínez and Chrispeels, 2003; Kamauchi et al., 2005). While DTT unbalances the redox environment in the ER and thereby prevents the formation of protein stabilizing disulfide bonds (Jämsä et al., 1994; Kamauchi et al., 2005), TM inhibits N-linked glycosylation, which is required for proper glycoprotein folding (Iwata and Koizumi, 2005). We found that NatB depleted plants were hypersensitive to DTT but not TM. This excludes a substantial contribution of NatB to the ER stress response and suggests that NatB mutants are susceptible to perturbations of the redox environment rather than the accumulation of unfolded proteins in the ER. Indeed, global transcriptome analyses revealed that DTT-responsive transcripts were induced in NatB mutants already under non-stressed conditions. In agreement with this finding, NatB mutants displayed lower reactive oxygen species (ROS) levels than wildtype plants. This pre-reduction of the cytosol might aggravate reductive stress upon DTT treatment. Furthermore, we demonstrate that *doa10* mutants were susceptible to neither DTT nor TM, ruling out an exclusive role of DOA10 in the plant ERAD. In summary, we provide further evidence that NatB-mediated imprinting of the proteome is vital for plants to respond to protein harming stress and exclude a role of DOA10 as the sole N-recognin for Ac/N-degron-containing NatB substrates in the plant ERAD pathway.

## Materials and Methods

### Plant Material and Growth Conditions

#### Growth on Soil

All work was performed with *Arabidopsis thaliana* ecotype Columbia-0 (Col-0). The T-DNA insertion lines *naa20* (SALK_027687), *naa25* (GK 819A05), *sdf2* (SALK_141321), and *doa10* (GK_588_A06.01) originate from the SALK and GABI-KAT collections (Alonso et al., 2003; Rosso et al., 2003). The NatA artificial microRNA (amiRNA) knockdown lines *amiNAA10* and *amiNAA15* were created by Linster et al. (2015). All experiments except the DTT and tunicamycin treatments (described below) were conducted with plants grown on soil (one half soil and one half substrate 2 from Klasmann-Deilmann, Germany) under short-day conditions (8.5 h light, 100 μE light photon flux density, 24°C/18°C day/night temperatures, and 50% humidity).

#### Growth on Sterile Medium

For growth on sterile medium, seeds were surface with 70% (v/v) ethanol (5 min) and 6% (v/v) NaClO (2 min) followed by three washing steps with sterile water. After 2 days of stratification at 4°C, the seeds were germinated under short-day conditions on solid 1x Murashige & Skoog (MS) medium (4 g/l MS-salts (Duchefa, Netherlands), 1% (w/v) sucrose, 0.4 g/l MES, and 0.7% (w/v) micro agar, pH 5.9).

### ER Stress Treatment

For long-term ER stress treatment, seeds were germinated under short-day conditions on solid 1×MS medium supplemented with 2–2.5 mM DTT or solid ½ MS medium supplemented with 50–100 ng/ml tunicamycin. To account for the delayed germination of *naa20* and *naa25*, seeds of these genotypes were pre-incubated for 24 h on control medium and subsequently transferred to fresh control or treatment plates.

To assess the effect of short-term reductive stress, seedlings were grown for 10 days on solid 1×MS medium. Afterward, they were transferred to liquid 1×MS medium (4 g/l MS-salts (Duchefa, Netherlands), 1% (w/v) sucrose, and 0.4 g/l MES, pH 5.9) supplemented with 10 mM DTT and incubated for 5 h. For the short-term tunicamycin treatment, six-day-old seedlings grown on solid ½ MS medium were transferred to liquid ½ MS medium containing 5 μg/ml tunicamycin or an equivalent volume of DMSO (control) and incubated for 5 h.

### Quantification of Chlorophyll Content

For the quantification of chlorophyll, 30 mg of mortared leaf material was mixed with 500 μl 95% ethanol, vortexed, and extracted at 70°C for 5 min. After centrifugation for 5 min, the supernatant was transferred to a new reaction tube and the extraction step was repeated. After combining both supernatants, the concentration of chlorophyll A (c_chla_) and chlorophyll B (c_chlb_) was determined photometrically at 664 nm (A_664_) and 649 nm (A_649_). To ensure correct measurement, the extracts were diluted with 95% ethanol to OD values between 0.2 and 0.9. The concentration of chlorophylls and carotenoids was calculated according to the following formulae (Lichtenthaler and Buschmann, 2001):


$$c_{\mathrm{chla}}\left(\mu g\;\;{\mathrm{ml}}^{-1}\right)=13,36\cdot A_{664}-5,19\cdot A_{649}$$

$$c_{\mathrm{chlb}}\left(\mu g\;\;{\mathrm{ml}}^{-1}\right)=27,43\cdot A_{649}-8,12\cdot A_{664}$$

### PCR

PCR for identification of T-DNA insertion lines was performed with the Taq-DNA Polymerase (New England Biolabs, M0267L). Genotyping of T-DNA insertion lines *naa20*, *naa25,* and *doa10* was conducted with specific primer combinations for the wildtype (NAA20_LP, NAA20_RP, NAA25_LP, NAA25_RP, DOA10_LP, and DOA10_RP) and mutant allele (SALK_BP and GK_BP). For cloning, DNA was amplified with the high-fidelity DNA polymerase Phusion (New England Biolabs, M0530L). All enzymes were used according to the supplier’s instructions manual. The corresponding primer sequences are listed in the Supplementary Table S6.

### Reverse Transcription Quantitative PCR (qRT-PCR)

To analyze *bZIP60* and *DOA10* transcript levels, total RNA was extracted from seedlings using the RNeasy Plant Kit (Qiagen, Germany). Subsequently, total RNA was transcribed into complementary DNA (cDNA) with the RevertAid H Minus First Strand cDNA Synthesis Kit using oligo(dT) primers (Thermo Scientific). All reactions were conducted according to the supplier’s protocol. The cDNA was analyzed by qPCR with the qPCRBIO SyGreen Mix Lo-ROX (PCR Biosystems) and PP2A (AT1G69960, Czechowski et al., 2005) as reference gene. The primer sequences for specific amplification of genes are listed in Supplementary Table S6. Data were analyzed via Rotor-Gene Q Series Software (v2.0.2).

### Determination of the Global Transcriptome

The peqGOLD Total RNA Kit (Peqlab) was used to extract RNA from 17-day-old wildtype and *naa20* seedlings grown on 1×MS medium with or without 2 mM DTT under short-day conditions. A global transcriptome analysis was performed using the GeneChip Arabidopsis Gene 1.0 ST Arrays from Affymetrix (High Wycombe, United Kingdom) as described in Linster et al. (2015). The arrays were annotated with a Custom CDF Version 16 with TAIR-based gene definitions. Quantile normalization and RMA background correction were applied to normalize the raw fluorescence intensity values. Differentially expressed genes were identified with the commercial software package SAS JMP10 Genomics, version 6, from SAS (SAS Institute, Cary, NC, United States). The applied false discovery rate correction allowed for a false-positive rate of alpha = 0.05 as the level of significance. Transcripts differentially regulated (>2-fold up- or downregulated, *p* < 0.05) in *naa20* and wildtype in response to DTT were functionally annotated. Overrepresented biological processes were identified based on the DAVID Bioinformatics Resources 6.8 gene ontology analysis (Huang et al., 2009). For this purpose, only processes with a gene count of ≥5 and a value of *p* < 0.05 were taken into account.

### Determination of ROS Levels

Reactive oxygen species levels were quantified in intact roots according to a modified version of the stomata-specific protocol by Pei et al. (2000). Six-week-old seedlings grown on ½ MS medium supplemented with 0.8% agarose under short-day conditions were shaken for 1 h in ½ MS liquid medium (pH 6.1) at 80 rpm. Subsequently, 5 mM H_2_DCFDA (2′,7′-dichlorodihydrofluorescein diacetate) was added to the medium and incubation was continued for 30 min. The roots were washed four times for 5 min in ½ MS medium. Then, the ROS levels were quantified using a confocal laser scanning microscope (Nikon C2, excitation: 488 nm, emission 525 nm). The fluorescence intensity was quantified using the software Fiji.

### Elemental Analysis of Rosette Leaves

The elemental analysis was carried out in cooperation with the Rothamsted Research Centre (UK). For this purpose, rosette leaves were dried for 4 days at 80°C and then ground to a fine powder. The mortars and pestles used were incubated overnight in 0.1 M HCl solution and then washed with ddH_2_O. Subsequently, 10 mg of material was transferred to glass vessels. Samples were incubated for at least 2 h at RT in 5 ml of an 85:15 (v/v) mixture of nitric acid (Primar, Aristar s.g = 1.42, 70%) and perchloric acid (Aristar/Primar, 70%). Subsequently, the sample was digested in a heating block (Carbolite, London, England) for 3 h at 60°C, 1 h at 100°C, 1 h at 120°C, and 1.5 h at 175°C. The ramping was 1°C/min for the first and last and 2°C/min for the second and third steps. Subsequently, 2 ml of a 25% (v/v) nitric acid (Primar, Aristar s.g = 1.42, 70%) was added to the mixture and heated at 80°C for 30 min. The extract was then diluted with ddH_2_O to a final volume of 10 ml and heated once more at 80°C for 30 min. Finally, the samples were cooled down to room temperature, the volume was refilled to 10 ml if necessary and analyzed by ICP-AES (Applied Research Laboratories, Vallaire, Ecublens, Switzerland).

### Basic Statistical Analysis

**Figure 1:**
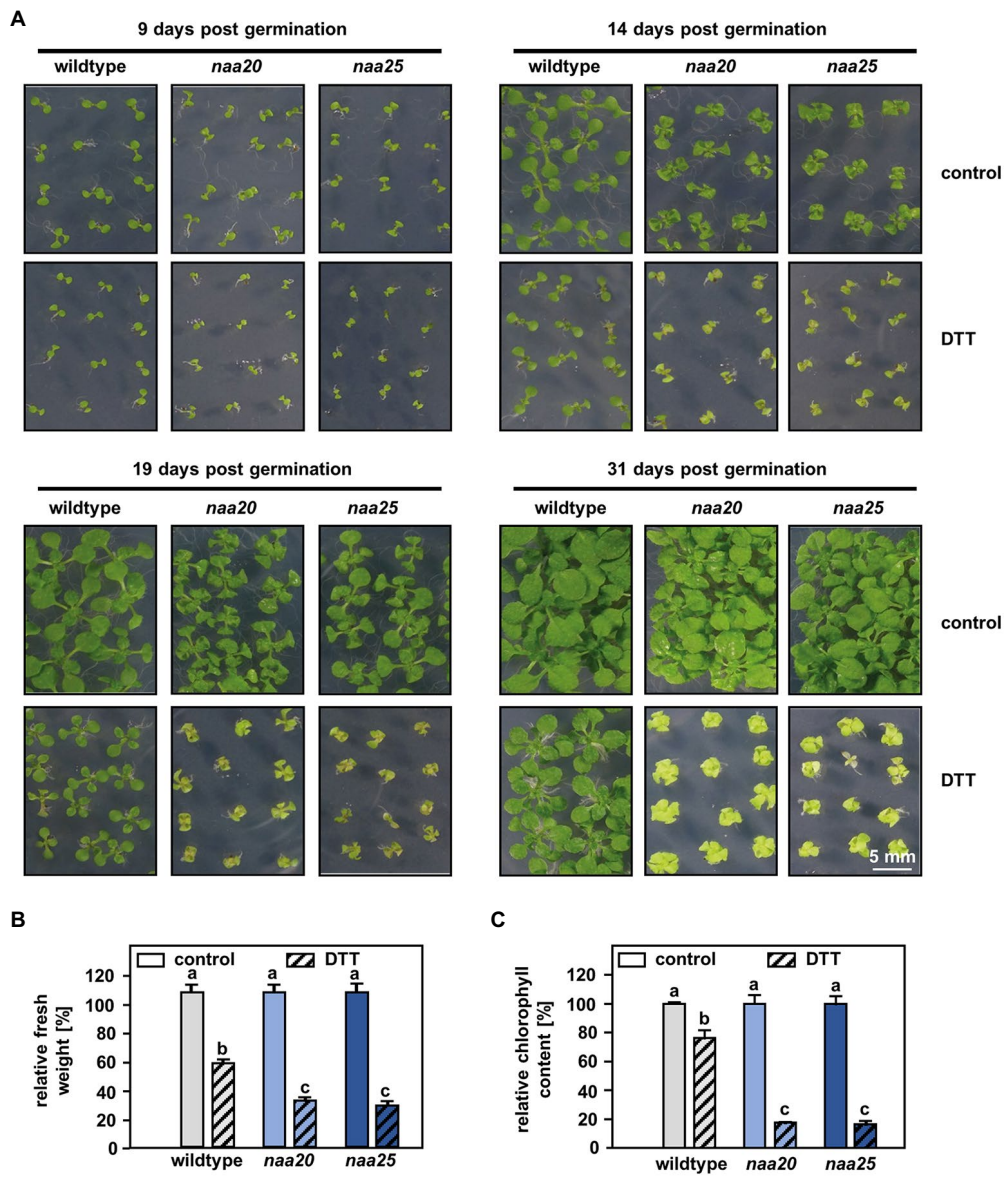
NatB mutants are sensitive to reductive stress. **(A)** Seeds of NatB mutants and wildtype plants were surface sterilized, stratified, and germinated on 1×MS medium. After 3 days of growth under short-day conditions, the seedlings were transferred to 1×MS plates (control) or 1xMS plates supplemented with 2 mM DTT (DTT). Pictures show the growth of seedlings at different time points post-germination (scale bar = 4 mm). The relative fresh weight **(B)** and relative chlorophyll content **(C)** of the plants were determined 31 days post-germination. Data given as means ± SE. Different letters indicate individual groups identified by pairwise multiple comparisons with a Holm-Sidak, One-way ANOVA (*p* < 0.05, n = 4, 1n = 5 seedlings).

**Figure 2:**
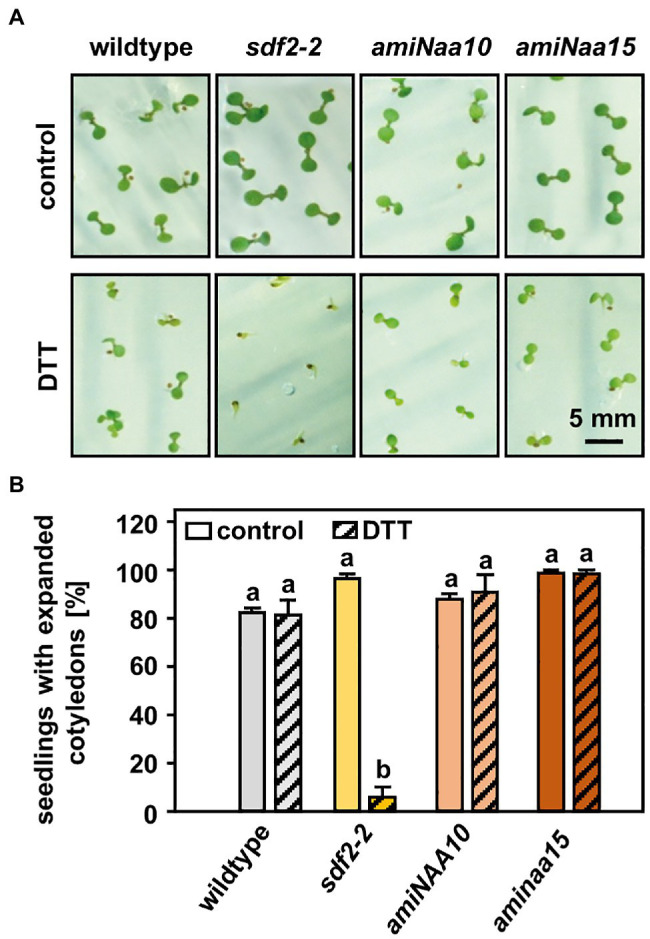
NatA mutants do not display an increased sensitivity to reductive stress. **(A)** Seeds of NatA mutants, the ER stress-sensitive positive control *sdf2-2* (*Arabidopsis stromal-derived factor 2*), and wildtype plants were surface sterilized, stratified, and germinated on 1×MS medium (control) or 1×MS medium supplemented with 2.5 mM DTT. Seedling was grown under short-day conditions as described in material and methods. Images were taken 10 days after stratification (scale bar = 5 mm). **(B)** Quantification of seedlings with expanded cotyledons. Data given as means ± SE. Different letters indicate individual groups identified by pairwise multiple comparisons with a Holm-Sidak, One-way ANOVA (*p* < 0.05, *n* = 3, 1n ≥ 20 seedlings).

**Figure 3:**
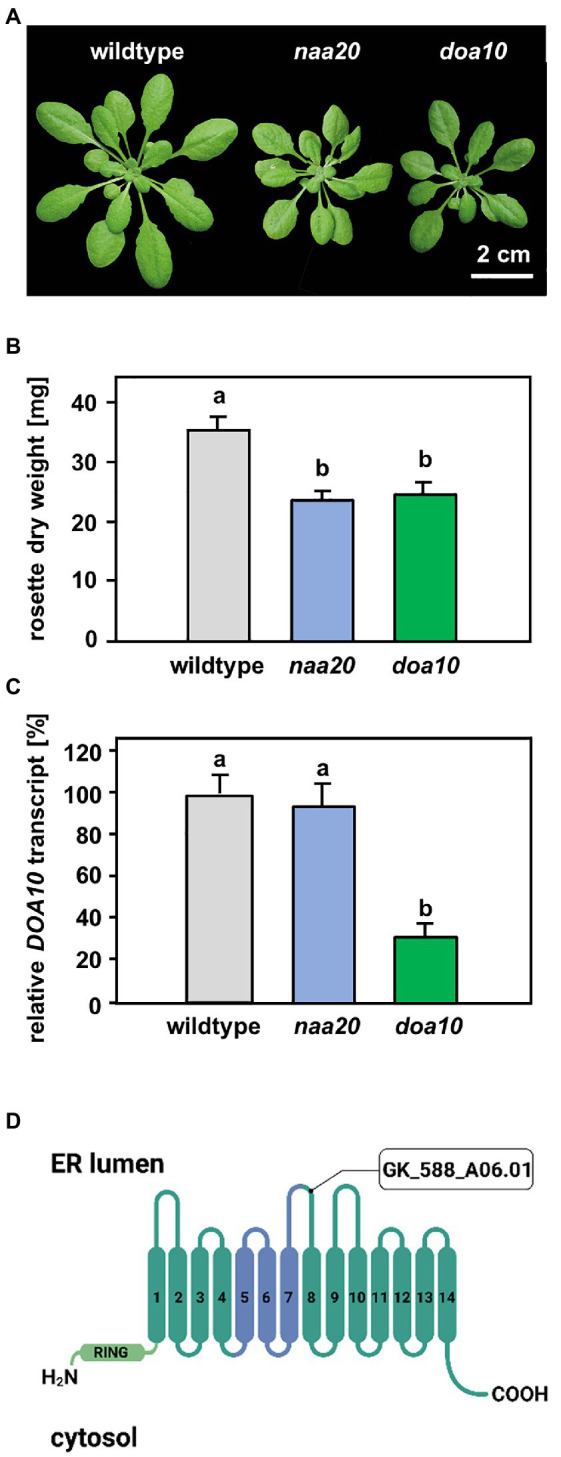
Comparison of *naa20* and *doa10* under optimal growth conditions. **(A)** Growth phenotype of representative wildtype, *naa20* and *doa10* plants after 6 weeks of growth on soil under short-day conditions (scale bar = 2 cm). **(B)** Rosette dry weight of six-week-old soil-grown wildtype, *naa20* and *doa10* plants. **(C)** Relative *DOA10* transcript level in ten-day-old seedlings (*n* = 3, 1n = 15 seedlings). The forward and reverse primer bound to the seventh and eight exons of the DOA10 transcript, respectively. **(D)** The tDNA insertion in GK_588_A06.01 disrupts the eighth of 14 transmembrane domains in the DOA10A protein. The conserved 130-residue domain present in all DOA10 orthologs (Swanson et al., 2001) is shown in blue, whereas the RING domain is represented in light green.

**Figure 4:**
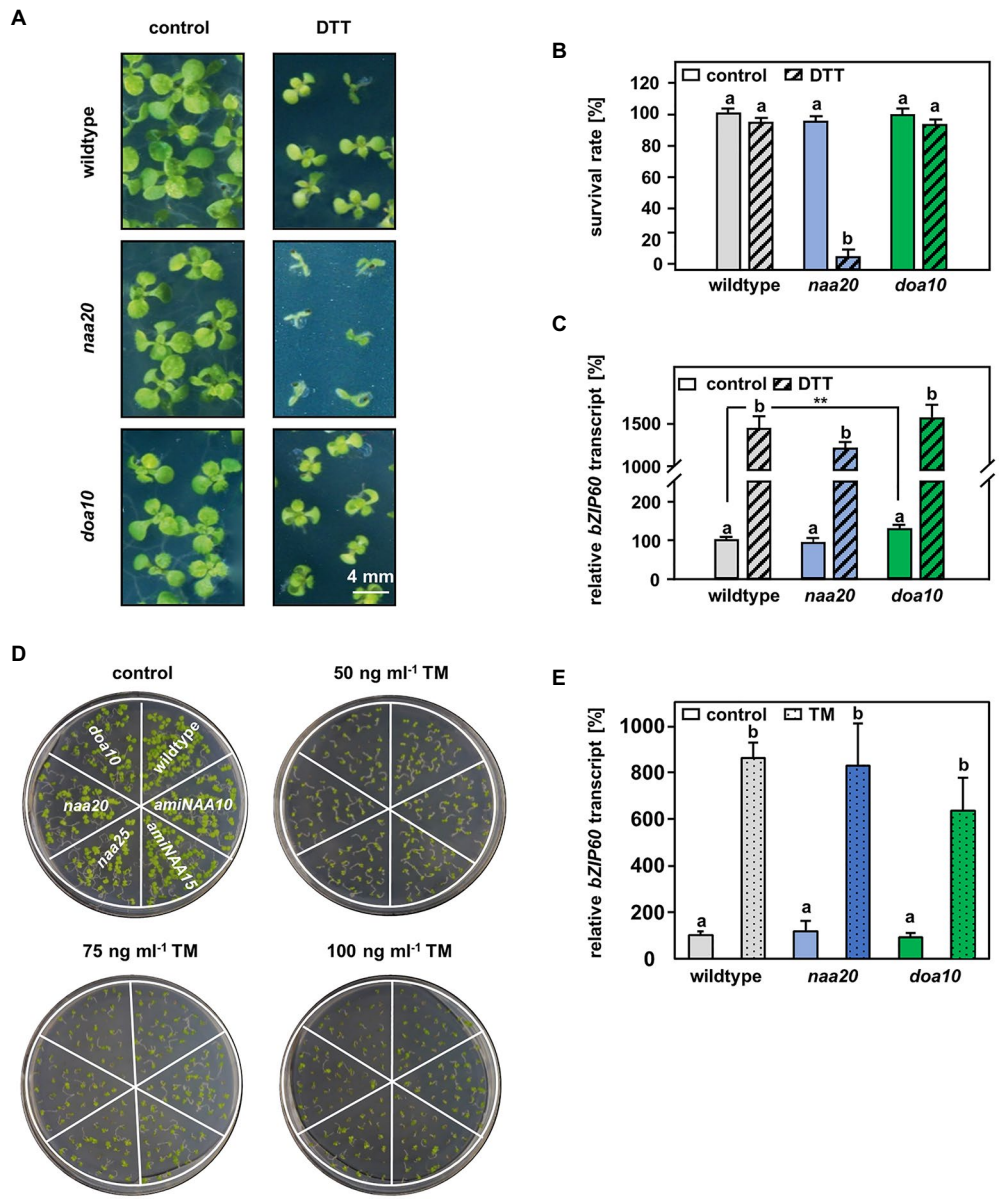
The connection between DOA10 and NAA20 mediated N-terminal acetylation. **(A)** Representative depiction of the seedlings after 3 weeks of growth on 1 x MS medium with and without 2.5 mM DTT (*n* = 3, 1n ≥29 seeds) (scale bar = 4 mm). **(B)** Quantification of living seedlings as shown in A. **(C)** Relative *bZIP60* transcript level in ten-day-old seedlings after 5 h of incubation in liquid 1 x MS medium (control) supplemented with 10 mM DTT (DTT). **(D)** Representative depiction of the seedlings after 10 days of growth on 1/2 x MS medium supplemented with 0, 50, 75, or 100 ng/ml tunicamycin (TM). **(E)** Relative *bZIP60* transcript level determined in six-day-old seedlings after 5 h of incubation in liquid ½ MS medium containing 5 μg/ml tunicamycin or and equivalent volume of DMSO (control). Data given as means ± SE. Different letters indicate individual groups identified by pairwise multiple comparisons with a Holm-Sidak, One-way ANOVA (*p* < 0.05).

Statistical analysis of the data presented in Figures 1B,C, 2B, 3B,C, 4B,C,E was conducted using SigmaPlot 12.0. Means from different sets of data were analyzed for statistically significant differences with the Holm-Sidak One-Way ANOVA test or the student’s *t*-test. Significant differences (*p* < 0.05) are indicated with different letters.

## Results

### Bioinformatical Analysis of Putative NatB Substrates and Their Subcellular Localization

Depletion of NatB results in enhanced sensitivity to protein harming conditions like high salt and osmolality (Huber et al., 2020). This finding prompted us to investigate the role of NatB in the response to ER stress. The ER is a major site for protein maturation. In the ER lumen, secretory and transmembrane proteins undergo posttranslational modifications, folding, and oligomerization until they acquire their mature form (Howell, 2013; Strasser, 2018). To estimate the number of NatB substrates maturing in the ER, we merged data retrieved from the eukaryotic subcellular localization database (eSLDB^1^) and the N-ter database.^2^ The eSLDB harbors information about 2,637 proteoforms with experimentally verified localizations. Of those, 1,079 (40.9%) localize to the ER, the secretory pathway, or the plasma membrane. Based on their N-terminal sequence, 638 (59.1%) of those proteoforms are putative NatA and 245 (22.7%) are putative NatB substrates (Supplementary Table S1). The N-ter database provides information about 2,206 proteoforms with experimentally verified acetylated N-termini (≥10% acetylation frequency). Of the 1,079 proteoforms localizing to the ER, the secretory pathway, or the plasma membrane according to eSLDB, 105 (9.7%) are annotated as experimentally verified Nat substrates in the N-ter database. Of those, 59 (56.2%) are putative NatA and 40 (38.1%) are predicted NatB substrates (Supplementary Table S1). Four of those putative NatB substrates (AT3G12800.1, AT2G13360.1, AT3G45780.1, and AT4G23400.1) have previously been identified as NatB substrates in Huber et al. (2020). In conclusion, a substantial number of putative NatB substrates localize to the ER or pass through the ER on their way to the plasma membrane or the extracellular matrix. This finding motivated us to analyze the performance of NatB mutants under DTT-induced ER stress.

### NatB but Not NatA Depletion Results in Hypersensitivity to DDT

For this purpose, seedlings of wildtype plants and the tDNA insertion lines *naa20* (SALK_027687) and *naa25* (GK-819A05) affected in the catalytic (NAA20, AT1G03150) or the auxiliary subunit (NAA25, AT5G58450) of the NatB complex (Ferrández-Ayela et al., 2013; Huber et al., 2020) were investigated. After 3 days of growth under short-day conditions, the seedlings were transferred to 1×MS plates (control) or 1×MS plates supplemented with 2 mM DTT. By 9 days post-germination, all three genotypes displayed visible growth reductions upon DTT treatment in comparison with the respective control groups (Figure 1A). The first differences between NatB mutants and wildtype plants became apparent 14 days post-germination when DTT-treated *naa20* and *naa25* showed signs of chlorosis. This effect further increased over the duration of the treatment (Figure 1A). While the DTT treatment resulted in an approximately 50% reduction in biomass (Figure 1B) and a 20% drop in chlorophyll content in wildtype plants (Figure 1C), NatB mutants suffered from a 70% reduction in biomass and an 80% decrease in chlorophyll content. To determine whether this enhanced sensitivity to DTT was exclusive to NatB mutants or merely a pleiotropic effect of impaired NTA at the ribosome, the NatA depleted lines *amiNAA10* and *amiNAA15* (Linster et al., 2015) were subjected to DTT stress (Figure 2A). The ER stress-sensitive mutant *sdf2-2* (Schott et al., 2010) was included as a positive control for the stress treatment. Ten days after germination on 2.5 mM DTT, the NatA mutants were affected in their development and morphology to a comparable extent as wildtype plants. While more than 90% of the *sdf2-2* mutants died during germination, wildtype and NatA mutants were still able to form cotyledons upon DTT exposure (Figure 2B). Taken together, these findings indicate that NatB, but not NatA, has a specific function in the response to DTT-induced protein harming stress.

### The E3 Ligase DOA10 Is Not Involved in the Adaptation to DTT-Induced Stress

A possible explanation for the increased sensitivity of NatB mutants toward protein harming stresses is the lack of unshielded Ac/N-degrons that allow for efficient degradation of misfolded NatB substrates via the UPS. In yeast, NatB-imprinted Ac/N-degrons are recognized by the ubiquitin E3 ligase DOA10 (Hwang et al., 2010). DOA10 predominantly targets proteins embedded in the ER- or nuclear membrane, but also soluble proteins of the cyto- and nucleoplasm (Ravid et al., 2006). Since DOA10 (AT4G34100) is conserved in Arabidopsis (Lü et al., 2012; Zhao et al., 2014), we compared *naa20* and *doa10* under non-stressed conditions and analyzed the interplay of NatB and DOA10 during ER stress in plants. Under non-stressed conditions, NatB mutants and *doa10* (GK_588_A06.01) plants displayed decreased rosette growth (Figure 3A) corresponding to a loss of 30% dry weight in comparison with wildtype plants (Figure 3B). The *doa10* mutant is a loss-of-function mutant since no full length mRNA can be amplified (Lü et al., 2012). However, the disruption of the *DOA10* gene in the fifth exon resulted in a 70% knockdown of a partial *DOA10* transcript. In the *naa20* background, relative *DOA10* transcript levels remained unaffected (Figures 3C,D). To investigate the role of DOA10 under DTT-induced ER stress, seeds of wildtype, *naa20* and *doa10* lines were grown for 3 weeks on 1×MS medium supplemented with 2.5 mM DTT (Figure 4A). All three genotypes suffered from a significant stress-induced growth inhibition. However, the seedlings of the wildtype and *doa10* survived this stress, suggesting that DOA10 has no substantial function in the plant ERAD pathway. In contrast, 90% of *naa20* died upon DTT treatment (Figure 4B). Based on these findings, we conclude that NatB has a specific function during the DTT-induced stress that is independent of DOA10.

### ER Stress Sensing Is Still Functional in NatB Depleted Mutants

To determine whether ER stress sensing was still intact in *naa20* and *doa10*, ten-day-old seedlings were treated with 10 mM DTT in 1×MS liquid medium for 5 h and the expression levels of the established ER stress-induced transcription factor basic leucine zipper 60 (bZIP60) were determined by qRT-PCR (Wang et al., 2012). Under control conditions, no significant differences between *bZIP60* levels in wildtype and *naa20* were detectable (Figure 4C), suggesting that the depletion of NatB does not result in a constitutive induction of the ER stress response. In agreement with previous reports, *doa10* mutants showed a 1.2-fold increased *bZIP60* baseline transcript level in comparison with wildtype plants (Lü et al., 2012). Upon treatment with DTT, all three genotypes displayed a more than 10-fold upregulation of the *bZIP60* transcript, indicating proper perception of ER stress.

### NatB Mutants Are Hypersensitive to a Perturbed Redox Homeostasis Rather Than ER Stress

In order to confirm that *naa20,* but not *doa10,* mutants are indeed sensitive to ER stress, tunicamycin (TM) was used as an alternative ER stress-inducing agent. TM inhibits N-linked glycosylation, which is required for proper glycoprotein folding (Iwata and Koizumi, 2005). Remarkably, *naa20* mutants were not hypersensitive to TM when grown on ½×MS medium supplemented with 50, 75, or 100 ng/ml of TM. Even though all three concentrations of the ER stress-inducing agent impaired growth of the tested genotypes, the TM-induced growth inhibition was comparable between wildtype, *naa20*, *doa10,* and the NatA mutants *amiNAA10* and *amiNAA15* (Figure 4D). The quantification of the relative *bZIP60* transcript levels in wildtype, *naa20* and *doa10* revealed that the three genotypes responded to TM exposure with an upregulated transcription of bZIP60 (Figure 4E). Taken together, those findings strongly suggest that DOA10 does not act as an N-recognin for Ac/N-degron containing NatB substrates in the plant ERAD pathway, or that only a few ER-resident NatB substrates possess an Ac/N-degron. Moreover, the experiments demonstrate that both, DTT and TM, induce ER stress in *naa20*, but only the reductive agent DTT elicits a hypersensitivity response. These results suggest that DTT-treated NatB mutants are rather hypersensitive to the perturbed cytosolic redox environment than the accumulation of unfolded proteins in the ER.

### NatB Mutants Respond Strongly to Reductive Stress on the Transcriptional Level

**Figure 5:**
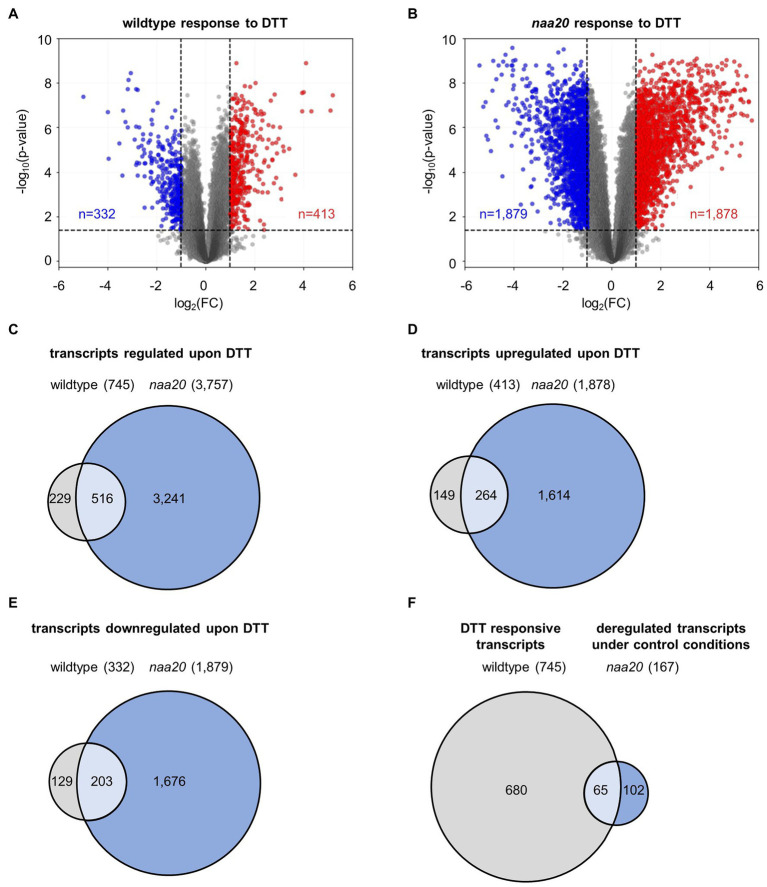
DTT-induced changes in the wildtype and *naa20* transcriptome. Total RNA was extracted from 17-day-old *naa20* and wildtype seedlings grown on 1 x MS medium with or without 2 mM DTT under short-day conditions (*n* = 3). The transcriptome was analyzed with an Affymetrix GeneChip (*n* = 27,826). **(A,B)** Volcano plot analyses of **(A)** wildtype and **(B)** *naa20* transcriptomes indicate the significantly upregulated (red) or downregulated transcripts (blue) upon DTT treatment (>2-fold; *p* < 0.05, indicated by dashed lines). **(C)** Venn diagram displaying the co-regulated transcripts in wildtype and *naa20* upon DTT treatment. **(D,E)** Overlap between co-induced **(D)** or co-repressed **(E)** transcripts in wildtype and *naa20*. **(F)** Venn diagram showing overlap of DTT-responsive transcripts in wildtype and constitutively deregulated transcripts in *naa20* under control conditions. The reductive stress response is constitutively active in NatB mutants. Of 167 transcripts which are deregulated in *naa20* under standard growth conditions, 65 (39%) are responsive to DTT.

The underlying mechanisms of the *naa20* hypersensitivity to reductive stress were investigated by performing a global transcriptome analysis on 17-day-old *naa20* and wildtype seedlings grown on 1×MS medium supplemented with 2 mM DTT (Supplementary Table S2, GEO identifiers GSE186324 (Reviewer Token: mxgpigeuhpoxror) and the super-series GSE186325 including GSE186324 and GSE132978). In wildtype, the expression of 745 of 27,826 analyzed transcripts was significantly (≥2-fold, *p* < 0.05) affected upon DTT treatment. Of those 745 transcripts, 332 were downregulated and 413 were upregulated (Figure 5A). Remarkably, the impact of DTT on transcription was more pronounced in *naa20* mutants, where 3,757 transcripts (1,878 up- and 1,879 downregulated) were differentially regulated (Figure 5B). An overlap of 516 transcripts which were regulated in both genotypes in response to DTT was identified (69% of regulated transcripts in wildtype / 14% of regulated transcripts in *naa20,* Figure 5C). Of those, only 49 were antagonistically regulated between the two genotypes (7% of regulated transcripts in wildtype/1% of regulated transcripts in *naa20,* Figures 5D–F). Of the 3,241 protein-coding transcripts which were specifically regulated in NatB in response to DTT, 739 (23%) encoded for putative NatB substrates. This compares well with the complete plant proteome which encompasses 25% predicted NatB substrates (Linster and Wirtz, 2018) and shows that the transcription of NatB substrates is not disproportionally affected by DTT treatment in NatB mutants.

**Figure 6:**
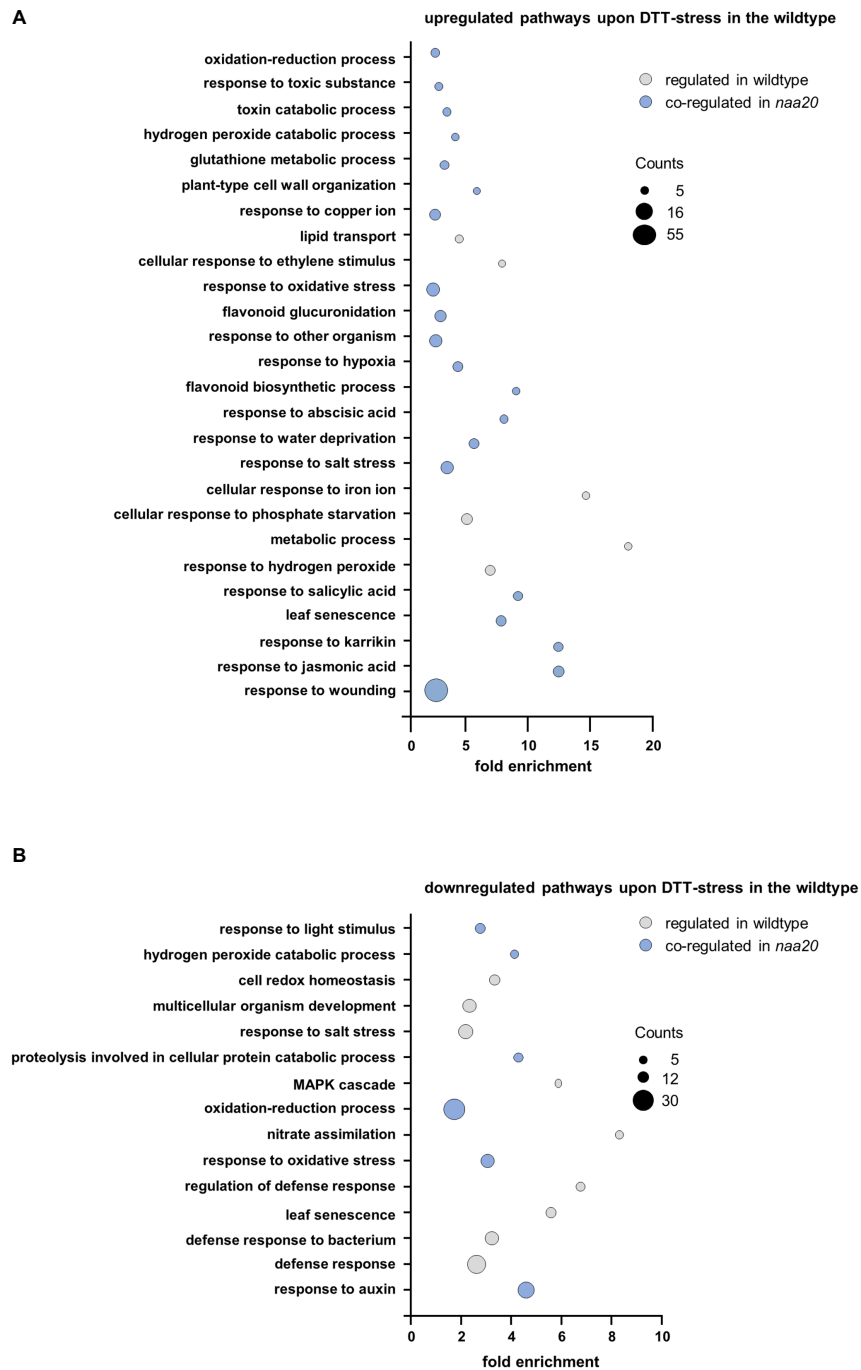
Gene ontology enrichment analysis of DTT-induced changes in the wildtype transcriptome. Total RNA was extracted from 17-day-old *naa20* and wildtype seedlings grown on 1 x MS medium with or without 2 mM DTT under short-day conditions (*n* = 3). The transcripts were analyzed *via* an Affymetrix GeneChip. Differentially regulated transcripts (>2-fold up- or downregulated compared to wildtype) were subjected to a gene ontology enrichment analysis performed with the DAVID Bioinformatics Resources tool v.6.8 (https://david.ncifcrf.gov/tools.jsp). Among the differentially regulated transcripts, genes involved in the depicted pathways were significantly (>2-fold, *p* < 0.05) enriched. The size of the bubbles corresponds to the number of regulated transcripts involved in the depicted pathways. The color code indicates pathways that were regulated in the same direction by DTT treatment in the wildtype and the *naa20* mutation (blue) or not co-regulated (gray). **(A)** Downregulated transcripts in wildtype upon DTT treatment. **(B)** Upregulated transcripts in wildtype upon DTT treatment.

The wildtype and *naa20* responses to reductive stress were dissected by a gene ontology (GO) enrichment analysis for significantly regulated genes (≥2-fold, *p* < 0.05) upon DTT treatment using the DAVID Bioinformatic Resources tool v. 6.8 (Figure 6,^3^ Supplementary Table S3). The 412 DTT-induced transcripts in wildtype were significantly enriched in 26 distinct biological processes (≥1.5-fold, involving at least five regulated genes, *p* < 0.05). Of those 26 processes, 20 (77%) were also upregulated by DTT treatment in *naa20* (Figure 6A). Similarly, the 332 transcripts found to be downregulated upon DTT exposure in WT were enriched in 15 biological processes of which 6 (40%) were downregulated in *naa20* as well (Figure 6B). Taken together those findings demonstrate that a substantial proportion of the reductive stress response was conserved between wildtype and *naa20*. This conserved response encompassed the induction of the non-enzymatic antioxidant machinery (e.g., flavonoid biosynthesis and glucuronidation) as well as further measures to maintain the cellular redox homeostasis (e.g., upregulation of glutathione metabolism and the response to hydrogen peroxide and hypoxia). In addition, DTT exposure triggered the response to several stress-related phytohormones (e.g., ABA, jasmonic acid, and salicylic acid) in both wildtype and *naa20*. For all three hormones, a direct involvement in the regulation of proteotoxic stress has already been demonstrated (Wang and Auwerx, 2017; Poór et al., 2019). Among the commonly downregulated processes, proteolysis involved in cellular protein catabolism as well as the response to the growth hormone auxin stood out. Auxins play an essential role in the coordination of plant growth and development. Altered redox conditions are known to perturb auxin signaling which in turn results in developmental defects (Bashandy et al., 2010).

Apart from those commonly regulated processes, the specific response of the wildtype to DTT included the upregulation of the responses to copper and iron. Since these trace elements catalyze Fenton reactions which generate hydroxyl radicals, excess of copper or iron induces oxidative stress and damages lipids, proteins, and even DNA (Lequeux et al., 2010; Thounaojam et al., 2012). In addition to the aforementioned processes which were regulated exclusively in wildtype plants, several processes involved in plant defense (e.g., against bacteria) were downregulated in wildtype and upregulated in *naa20* in response to DTT.

Whereas the wildtype response to DTT was largely conserved in *naa20*, many additional processes were perturbed in NatB depleted mutants upon reductive stress. Among the 1,878 transcripts upregulated in response to DTT in *naa20*, transcripts involved in 88 distinct biological processes were significantly enriched. Of those, 20 (23%) were similarly regulated in wildtype. The processes induced by DTT exclusively in *naa20* include the response to cadmium, the response to ROS, and the endoplasmic reticulum UPR. Since cadmium triggers ROS production in plants and causes protein oxidative damage (Perez-Chaca et al., 2014), those three processes are often co-regulated. Interestingly, in *naa20* transition metal ion, transport was already upregulated under unstressed conditions and resulted in a mild accumulation of copper, zinc, and nickel (Supplementary Figure S1). Yet another process which is upregulated exclusively in *naa20* upon DTT exposure is the response to the phytohormone ethylene which regulates various aspects of UPR (Depaepe et al., 2021).

The most striking difference between the response to DTT in wildtype and *naa20* was however the extensive upregulation of plant defense mechanisms (e.g., against fungi, nematodes, viruses, bacteria, oomycetes, and insects) which was exclusive to *naa20*. Of 88 upregulated processes in *naa20*, 30 (34%) were directly involved in plant biotic stress responses. In addition, 64 processes were exclusively downregulated in *naa20* as a consequence of the DTT treatment. Those processes can be categorized as processes implicated in transport (e.g., of lipids, oligopeptides, and amino acids), the response to light (e.g., red and blue light and the regulation of photosynthesis via the organization of chloroplast and chlorophyll biosynthesis) and cell wall organization (e.g., cell wall biogenesis and loosening as well as pectin, lignin, and cellulose catabolic processes). The reorganization of the cell wall was accompanied by a downregulation of developmental processes (leaf vascular tissue formation, trichome branching, and xylem development). This was well in line with a downregulation of gibberellic acid and cytokinin-mediated signaling pathways, as well as brassinosteroid biosynthesis. While gibberellic acid and cytokinin stimulate plant growth, brassinosteroids in particular control the division, expansion, and differentiation of several cell types (Gupta and Chakrabarty, 2013; Oh et al., 2020).

### The Response to Reductive Stress Is Constitutively Activated in NatB Mutants

Interestingly, out of the 167 transcripts differentially expressed between wildtype and *naa20* under standard growth conditions (Huber et al., 2020), 65 (39%) belong to the group of DTT-responsive transcripts in the wildtype identified in this study (Figure 5F, Supplementary Table S2). Furthermore, the gene ontology enrichment analysis revealed that the response to oxidative stress was downregulated in *naa20* (Huber et al., 2020) and became even further depleted upon DTT treatment (Supplementary Table S3). Both findings hint toward a constitutively active reductive stress response in *naa20*. Interestingly, the expression of cytosolic chaperones was not elevated in *naa20* under standard growth conditions as would be expected for continuously stressed plants (Supplementary Table S4). Only after DTT exposure, *naa20* specifically upregulated the transcription of the cytosolic chaperone HSC70-1 (AT5G02500) and the ER-localized chaperone BIP1 (AT5G28540).

### The Cytosol of NaB Mutants Is Over-Reduced Under Standard Growth Conditions

**Figure 7:**
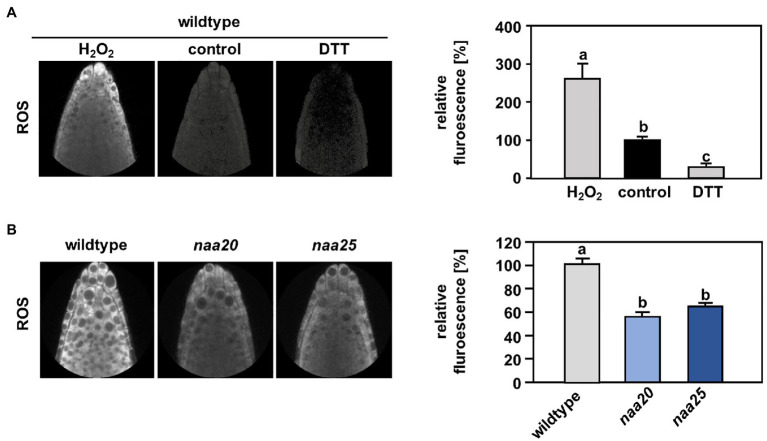
The cytosol of NatB mutants is more reduced than in the wildtype. To determine the reactive oxygen species (ROS) level in the cytosol of roots cells, wildtype seedlings were grown on ½ MS medium under short day conditions. After six days, the plants were transferred to liquid ½ MS medium supplemented with the ROS indicator H_2_DCFDA (2′,7′-dichlorodihydrofluorescein diacetate), and the ROS-triggered fluorescence intensity was determined by non-invasive confocal laser scanning microscopy as described in material and methods. **(A)** Representative images of wildtype roots treated for 30 min with water (control, 100 %) or water supplemented with either 100 mM H_2_O_2_ or 10 mM dithioerythritol (DTT). The relative ROS-triggered fluorescence upon H_2_O_2_ and DTT treatment was quantified with respect to control conditions in >5 roots. **(B)** Representative fluorescence images of roots from the wildtype and plants depleted of the catalytic (*naa20*) or the auxiliary NatB subunit (*naa25*) treated with H_2_DCFDA. The relative ROS-triggered fluorescence in *naa20* and *naa25* was quantified with respect to the wildtype in >5 roots. Data given as means ± SE. Different letters indicate individual groups identified by pairwise multiple comparisons with a Holm-Sidak, One-way ANOVA (*p* < 0.05, *n* ≥ 5).

Reductive stress reduces ROS levels to below physiological concentrations and thereby perturbs the signaling function of ROS (Xiao and Loscalzo, 2020). In order to determine whether NatB mutants suffer from constitutive reductive stress, the level of ROS was measured in the roots of wildtype and *naa20* seedlings under standard growth conditions by application of the ROS indicator H_2_DCFDA. To provide a functional proof that H_2_DCFDA can sense redox perturbation in the cytosol of intact roots, we treated seedling with a combination of the ROS indicator and either H_2_O_2_ or DTT. As expected, H_2_O_2_ caused a significant increase of H_2_DCFDA-dependent fluorescence, whereas DTT triggered decreased fluorescence when compared to control conditions (Figure 7A). Indeed, *naa20* seedlings displayed lowered ROS levels in comparison with wildtype plants (Figure 7B), indicating an over-reduction of the *naa20* cytosol under standard growth conditions. This over-reduction might explain the constitutive induction of the reductive stress response in *naa20* observed on the transcript level. Whereas this over-reduction might not be detrimental under normal circumstances, it could aggravate the protein harming effects of DTT exposure, resulting in the observed hypersensitivity of NatB depleted plants to the reductive agent.

## Discussion

While for individual proteins, NTA has been shown to affect protein turnover, folding, and subcellular localization, the overall significance of NTA is still controversially discussed (Aksnes et al., 2016). In 2011, a bioinformatic analysis revealed that cytosolic proteins are mostly N-terminally acetylated, whereas proteins passing through the ER typically harbor free N-termini. This trend was more pronounced in yeast than in humans and plants. The authors of the study concluded that in eukaryotes, NTA inhibits targeting of proteins to the ER in an evolutionary conserved manner (Forte et al., 2011). Following this rationale, a lack of NatA or NatB, which acetylate up to 60% of the plant proteome, could lead to a massive accumulation of non-acetylated cytosolic Nat substrates in the ER. Such an accumulation of mis-targeted proteins would likely result in constitutive ER stress. In this study, we investigated the consequences of NatB depletion for the ER stress response. By assessing the transcript levels of the ER stress marker *bZIP60* under standard growth conditions, we revealed that NatB mutants did not suffer from constitutive ER stress (Figures 4C,E). This finding suggests that the depletion of NatB does not result in an accumulation of mis-sorted non-acetylated NatB substrates in the ER. In addition, we identified a significant number of N-terminally acetylated putative NatA (59) and NatB (40) substrates which localize to the ER, the plasma membrane, or the secretory pathway, demonstrating that NTA does not generally inhibit ER targeting of proteins. However, we previously demonstrated that NatB depleted mutants were sensitive to diverse protein harming stresses. These findings motivated us to analyze the function of NatB during ERAD. ERAD is a consequence of protein misfolding that would lead to unshiedling of NatB-imprinted Ac/N-degron recognized by DOA10. To investigate the interplay of DOA10 and NatB in the ER stress response, seedlings were subjected to the ER stress-inducing agents DTT or tunicamycin (TM).

We found that depletion of DOA10 did not result in hypersensitivity to DTT (Figure 4A) or TM (Figure 4D). In yeast, DOA10 targets misfolded proteins in the ER for degradation via the proteasome (Bays et al., 2001; Swanson et al., 2001). In *Arabidopsis thaliana*, two DOA10 homologs termed DOA10A (AT4G34100) and DOA10B (AT4G32670) have been identified. Phylogenetic studies suggest that DOA10B is a Brassica-specific paralog which has diverged and likely neofunctionalized. Since heterologous expression of DOA10A, but not DOA10B, compensates for the loss of DOA10 function in yeast (Etherington et al., in preparation) we used DOA10A depleted plants in this study. Unless redundant enzymes with overlapping functions degrade DOA10A substrates during ER stress, our findings rule out a substantial contribution of *At*DOA10A to the degradation of proteins during ER stress and suggest a different role for DOA10A in plants. Indeed, *At*DOA10A has so far mainly been associated with the negative regulation of ABA biosynthesis in seeds (Zhao et al., 2014), the maintenance of the plant water status (Lü et al., 2012) and cuticular wax formation where it acts together with HIRD1. How the two potential ERAD-associated E3 ligases co-regulate wax biosynthesis is still under investigation (Wu et al., 2021).

Furthermore, neither NatA nor NatB depleted mutants were sensitive to ER stress induced by the N-glycosylation blocker TM (Figure 4D), excluding a substantial contribution of NatA or NatB to the ER stress response. On the contrary, NatB depleted plants were hypersensitive to the reductive agent DTT that can also induce ER stress (Figure 1). These findings suggest that NatB mutants are susceptible to alterations of the cellular redox environment rather than the accumulation of unfolded proteins during ERAD. In line with this hypothesis, NatB mutants displayed lower cytosolic ROS levels than wildtype plants under standard growth conditions indicating an over-reduction of the cytosol in NatB mutants. Also in humans, depleted ROS levels are a hallmark of pathophysiological situations (e.g., specific subtypes of Alzheimer’s disease) in which the cell becomes more reduced than in the normal, resting state (Lloret et al., 2016).

The cellular redox homeostasis is maintained by a set of non-enzymatic small molecule effectors (e.g., glutathione, ascorbic acid, or flavonoids) and electron-transferring enzymes (e.g., catalase, superoxide dismutase, or ascorbate peroxidase) which synergistically scavenge ROS (Huang et al., 2019). Both systems might contribute to the observed over-reduction of the cytosol in *naa20* seedlings. Out of the 246 enzymes directly involved in ROS metabolism (Oliveira et al., 2019), 49 (20%) are predicted NatB substrates (Supplementary Table S5). Aside from those direct candidates, any enzyme involved in the synthesis of the aforementioned non-enzymatic small molecule effectors or any transcription factor controlling the expression of the latter could contribute to the likely multifactorial over-reduction of the cytosol in *naa20*.

While it is difficult to pinpoint the cause of the redox imbalance in *naa20*, its consequences are readily observed on the transcript level. A global transcriptome analyses demonstrated that in comparison with wildtype plants, the response to ROS is downregulated in *naa20* already under normal growth conditions (Huber et al., 2020) and decreases further upon DTT treatment. In addition, the over-reduction of the cytosol in NatB mutants might explain certain striking differences between wildtype and *naa20* in the transcriptional response of NPR1-induced genes. NPR1 is a key regulator of the pathogen response and forms cytosolic oligomers stabilized through redox-sensitive intramolecular disulfide bonds. Changes in the cytosolic redox state lead to a reduction of the disulfide bonds in the NPR1 complex (Kinkema et al., 2000; Tada et al., 2008). In consequence, NPR1 monomers are released and translocated to the nucleus where they interact with basic leucine zipper transcription factors which induce the expression of a subset of genes (Blanco et al., 2005; Backer et al., 2019), including PR1 (AT2G14610), PR4 (AT3G04720), WAK1 (AT1G21250), and CRT3 (AT1G08450). Interestingly, the transcription levels of the aforementioned genes were unchanged in wildtype plants upon the here applied DTT concentration, while the same genes were 2.4- to 121-fold induced in *naa20*, suggesting that reductive stress-induced NPR1 monomers were only formed in *naa20*.

In summary, our results show that NatB-mediated imprinting of the proteome is vital for plants to respond to protein harming stress in the cytosol and exclude a major role of DOA10 as N-recognin for in the plant ERAD pathway.

## Data Availability Statement

The original contributions presented in the study are publicly available. This data can be found at: https://www.ncbi.nlm.nih.gov/geo/query/acc.cgi?acc=GSE186324, accession number PRJNA773404.

## Author Contributions

MH characterized the NatB DTT response. CD performed the global transcriptome analysis. LA evaluated the microarray data and performed the bioinformatical analysis on NatB substrates in the ER. RE performed the tunicamycin experiments. DG supervised and planned the tunicamycin experiments. MJH performed the elemental analysis of wildtype and *naa20*. MW and RH conceived and directed the study. MW and LA wrote the manuscript. All authors contributed to the article and approved the submitted version.

## Funding

Research at Heidelberg was funded by the German Research Council (DFG) via the Collaborative Research Centre 1036 (TP 13 to RH and MW), and the ERA-CAPS Research network “KatNat” (WI 3560/4-1 for MW). Research at Birmingham was funded by the Biotechnology and Biological Sciences Research Council (grant number: BB/M020568/1) and a European Research Council Starting Grant (715441-GasPlaNt) to DG. Rothamsted Research receives grant-aided support from the Biotechnology and Biological Sciences Research Council (BBSRC) through the Designing Future Wheat programme [BB/P016855/1].

## Conflict of Interest

The authors declare that the research was conducted in the absence of any commercial or financial relationships that could be construed as a potential conflict of interest.

## Publisher’s Note

All claims expressed in this article are solely those of the authors and do not necessarily represent those of their affiliated organizations, or those of the publisher, the editors and the reviewers. Any product that may be evaluated in this article, or claim that may be made by its manufacturer, is not guaranteed or endorsed by the publisher.

## Footnotes

^1^ http://gpcr.biocomp.unibo.it/esldb/download.htm

^2^ https://n-terdb.i2bc.paris-saclay.fr/exportData

^3^ https://david.ncifcrf.gov/
