# Supplementary material for: Disruption of the Nα-Acetyltransferase NatB Causes Sensitivity to Reductive Stress in Arabidopsis thaliana
Source: Front Plant Sci. 2022 Jan 3;12:799954. doi: 10.3389/fpls.2021.799954 (PMC8761761; doi:10.3389/fpls.2021.799954)
Supplement: Supplementary file 1 [file Data_Sheet_1.PDF]

Supplemental Figures Huber et al., 2021

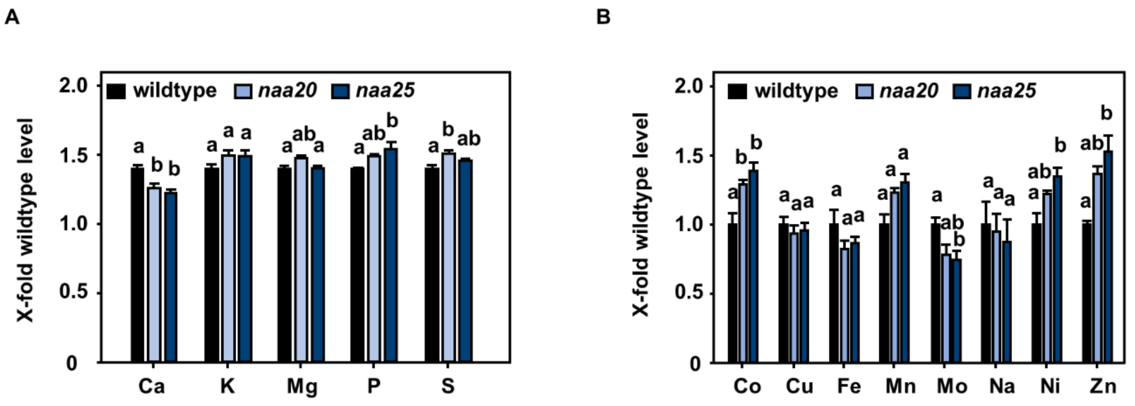

Supplemental Figure 1: Multi-element analysis of rosette leaves reveals disparities between NatB mutants and wildtype plants. Rosette leaves of six-week-old plants grown on soil under short day conditions were dried for four days at 80 °C. Afterwards the leaf material was ground and elements were extracted. The quantification was performed by ICP-AES. **A)** Macroelements. **(B)** Microelements. Data given as means ± SE. Different letters indicate individual groups identified by pairwise multiple comparisons with a Holm-Sidak, One-way ANOVA ($p<0.05$, $n \geq 3$).

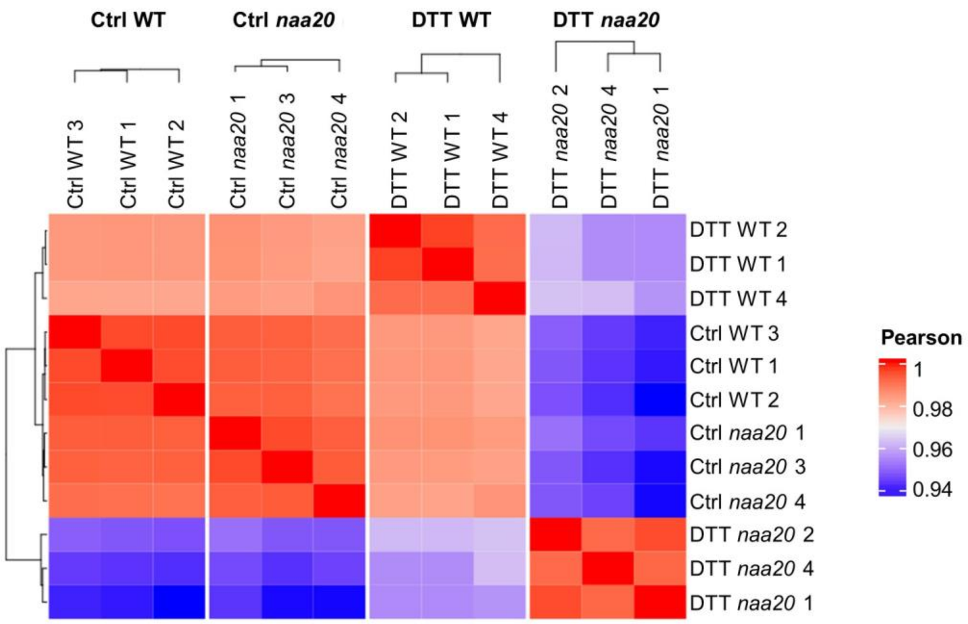

Supplemental Figure 2: Cluster analysis of transcriptome data by Pearson correlation.

Pearson correlation heatmap illustrating the cluster analysis of transcriptome data derived from DTT-treated and non-treated (Ctrl) wildtype (WT) and NatB depleted mutants (*naa20*). The correlation heatmap was created using the complexHeatmap package vers. 2.8.0 as described in Gu et al. (2016). Total RNA was extracted from four individual plants per condition. Based on mRNA integrity and purity, three samples were selected for transcriptome analysis (n=3).

Supplemental Reference:

Gu, Z., Eils, R., and Schlesner, M. (2016). Complex heatmaps reveal patterns and correlations in multidimensional genomic data. Bioinformatics **32,** 2847-2849.
